# Current and Future Use of Artificial Intelligence in Electrocardiography

**DOI:** 10.3390/jcdd10040175

**Published:** 2023-04-17

**Authors:** Manuel Martínez-Sellés, Manuel Marina-Breysse

**Affiliations:** 1 Cardiology Department, Hospital General Universitario Gregorio Marañón, Calle Doctor Esquerdo, 46, 28007 Madrid, Spain; 2 Centro de Investigación Biomédica en Red—Enfermedades Cardiovasculares (CIBERCV), Instituto de Salud Carlos III, 28029 Madrid, Spain; m@idoven.ai; 3 Facultad de Ciencias de la Salud, Universidad Europea, Villaviciosa de Odón, 28670 Madrid, Spain; 4 Facultad de Medicina, Universidad Complutense, 28040 Madrid, Spain; 5 IDOVEN Research, 28013 Madrid, Spain; 6 Centro Nacional de Investigaciones Cardiovasculares Carlos III (CNIC), Myocardial Pathophysiology Area, 28029 Madrid, Spain

**Keywords:** artificial intelligence, machine learning, deep learning, electrocardiography, diagnosis, prognosis, cost effectiveness

## Abstract

Artificial intelligence (AI) is increasingly used in electrocardiography (ECG) to assist in diagnosis, stratification, and management. AI algorithms can help clinicians in the following areas: (1) interpretation and detection of arrhythmias, ST-segment changes, QT prolongation, and other ECG abnormalities; (2) risk prediction integrated with or without clinical variables (to predict arrhythmias, sudden cardiac death, stroke, and other cardiovascular events); (3) monitoring ECG signals from cardiac implantable electronic devices and wearable devices in real time and alerting clinicians or patients when significant changes occur according to timing, duration, and situation; (4) signal processing, improving ECG quality and accuracy by removing noise/artifacts/interference, and extracting features not visible to the human eye (heart rate variability, beat-to-beat intervals, wavelet transforms, sample-level resolution, etc.); (5) therapy guidance, assisting in patient selection, optimizing treatments, improving symptom-to-treatment times, and cost effectiveness (earlier activation of code infarction in patients with ST-segment elevation, predicting the response to antiarrhythmic drugs or cardiac implantable devices therapies, reducing the risk of cardiac toxicity, etc.); (6) facilitating the integration of ECG data with other modalities (imaging, genomics, proteomics, biomarkers, etc.). In the future, AI is expected to play an increasingly important role in ECG diagnosis and management, as more data become available and more sophisticated algorithms are developed.

## 1. Introduction

Artificial intelligence (AI) refers to the idea of a computer model that makes decisions using a priori information and improves its performance with experience [1]. In the current manuscript, AI is used as a synonym of machine (deep) learning (algorithms) (hybrid) convolutional neural network. These terms do not mean exactly the same. For instance, machine learning is a sub-field of AI that uses computer algorithms to extract patterns from raw data, acquire knowledge without human input, and apply this knowledge for various tasks [2]. However, for simplicity, we will only use AI in our comprehensive review. Moreover, different models and algorithms have been used in the studies that we try to summarize in this comprehensive review. Readers should be aware that when we use the AI concept, we are including a very wide range of methodologies that have used several types of data, hardware, and software [3]. Moreover, new methods and techniques are continuously appearing, for instance, the recently developed ability to simultaneously perform beat detection, beat classification, and rhythm detection/classification has improved performance over previous algorithms [4]. Incorporating AI-based ECG algorithms can be difficult due to a range of organizational and regulatory hurdles that might make it difficult to develop solutions that are generalizable, interpretable, and user-focused [5].

Cardiovascular diseases (CVD) are the leading cause of death worldwide and have a particularly strong impact among patients with other comorbidities, such as diabetes mellitus or chronic kidney disease [6]. Their prevention, early diagnosis, and management are one of the most significant challenges in today’s world. AI might be used even before acquiring an electrocardiogram (ECG). For instance, in emergency departments, AI-based models can effectively predict whether patients presenting at triage will require an ECG [7]. Once ECG data are available, AI combines high accuracy with the ability to provide interpretation for the decisions made, opening the door for an overall clinical analysis and diagnosis [8]. AI also shortens the data processing time, provides real-time information [9], and saves a significant amount of time for the clinicians [10]. For instance, AI analyses of insertable cardiac-monitor-detected episodes is associated with high classification accuracy and reduces health care staff workload by triaging relevant data [11].

In 1970, before the authors of this manuscript were born, an AI-based model that analyzed ventricular repolarization abnormalities showed a high correlation with serum potassium levels [12]. Since then, AI algorithms that use ECG and patients’ data have been shown to improve different aspects of CVD [13]. AI is suitable to identify abnormal ECG [14] and provides a tool for the diagnosis of CVD that is increasingly used to assist in the diagnosis, risk stratification, and therapy guidance. The main areas where AI-based algorithms that use ECG data can help clinicians are shown in Table 1.

## 2. Interpretation/Detection of ECG and Cardiac Abnormalities

The basis of the detection of ECG abnormalities and arrhythmia diagnosis is the identification of normal versus abnormal individual heart beats and their correct classification into different diagnoses according to ECG morphology. It is challenging and time-consuming to distinguish these heartbeats on ECG and even more in long-duration ambulatory and Holter monitoring as these signals are typically corrupted by noise. AI-powered ECG interpretation has shown promising results, improving detection of arrhythmias, ST-segment changes, QT prolongation, and other ECG abnormalities. In addition, AI can also detect cardiac structural damage, such as myocardial hypertrophy or left ventricular systolic dysfunction.

### 2.1. Arrhythmias

Arrhythmia detection is one of the fields where AI has better shown its value, enabling multi-class arrhythmia detection [15] with impressive accuracies above 99% in controlled test datasets [16,17]. AI can use the variable length heart beats for arrhythmia detection with a high classification accuracy of 98% [18]. Automated ECG-interpreting software accurately detects arrhythmias, such as atrial fibrillation [19]. Attia et al. [20] showed that an AI-enabled ECG acquired during normal sinus rhythm permitted identification at point of care of individuals with atrial fibrillation with an area under the curve of 0.9 and an overall accuracy of 83%. Jo et al. [21] describe an average area under the receiver operating characteristic curve using a 12-lead ECG for arrhythmia classification of 0.98. AI is already outperforming cardiologists in arrhythmia detection and classification. Hannun et al. [22] reported an AI algorithm average area under the receiver operating characteristic curve of 0.97. The average F1 score (0.84) exceeded that of average cardiologists (0.78). The sensitivity of AI algorithm exceeded the average cardiologist sensitivity for all rhythm classes. In fact, we can assume that the accuracy of arrhythmia detection with AI-based models is already superior to the average cardiologist. Chang et al. [23] found that the accuracy for the classification of 12 heart rhythms of the AI model (0.90) was superior to the mean accuracies of internists (0.55), emergency physicians (0.73), and cardiologists (0.83). AI better or comparable performance to cardiologists has been shown not only for rhythm but also for conduction, chamber diagnosis, myocardial infarct, and other diagnoses [24]. Ribeiro et al. [25] found that AI outperformed cardiology resident medical doctors in recognizing six types of abnormalities in 12-lead ECG recordings with F1 scores above 80% and specificity over 99%. There is a wide range of arrhythmias in which both AI sensitivity and specificity are higher than those achieved by state-of-the-art classifiers [26], and AI can already identify 27–30 ECG abnormalities accurately based on various lead combinations of ECG signals [27,28]. AI is more accurate than physicians working in cardiology departments at distinguishing a range of distinct arrhythmias in single-label and multi-label ECGs [27].

**Figure 1:**
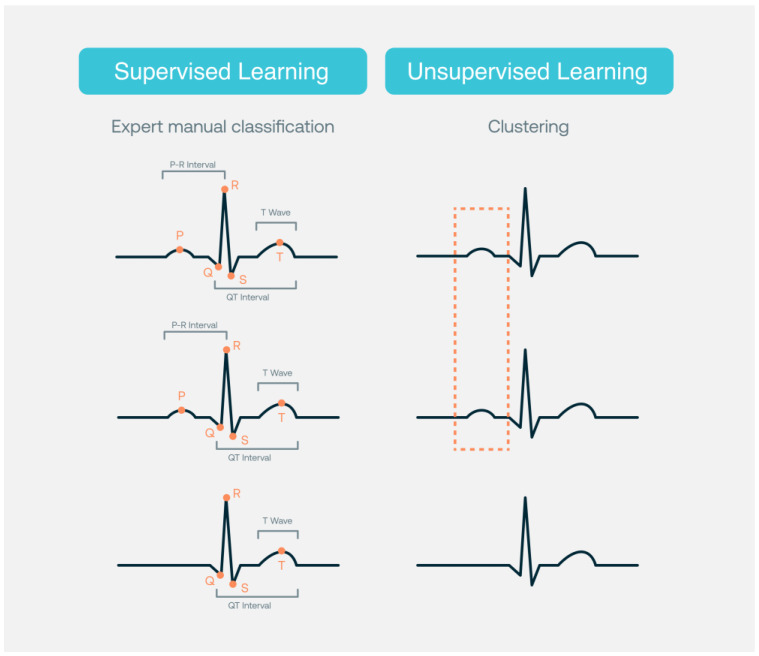
Supervised and unsupervised learning.

The AI-based strategy for the analysis of Holter recordings is faster and at least as accurate as a conventional analysis by electrophysiologists [29]. Acharya et al. [30] reported an accuracy of 94% in the diagnostic classification of heartbeats. Although arrhythmias detection and classification using off-the-shelf wearable devices, smartwatches, sport bands, and others is feasible [31], some devices and models might need physician overview to achieve clinically sufficient diagnostic accuracy for detection of some arrhythmias, such as atrial fibrillation [32]. In fact, human expert ECG overreading remains important in many clinical settings [33]. Figure 1 shows how supervised learning strategies require datasets annotated by cardiology experts for training, testing, and validation, while unsupervised learning strategies are based on clustering. The level of detail in the annotation process may vary (such as beginning and end of the QRS, QT interval, higher T-wave amplitude). This annotation is used by the model as guidance to “learn” what is the expected output. Unsupervised learning identifies visible or hidden data patterns from an unlabeled dataset. In unsupervised learning, the AI model is trained only on the inputs without ECG waveform annotations performed by cardiology experts.

AI detection of ventricular arrhythmia has achieved an accuracy, sensitivity, and specificity of 99.2–98.8% [34], which is comparatively better than the results of the standard classifier. Moreover, AI analysis of surface ECG facilitates the localization of idiopathic ventricular arrhythmias, which might optimize the ablation strategy [35]. The detection of lethal ventricular arrhythmia in automated external defibrillators with AI algorithms is under the focus of several studies. However, a few of them have used real-life, out-of-hospital cardiac arrest databases and applied deep learning algorithms in the two main environments of clinical use: regular automated external defibrillators analysis for shock advice in ECG without noise [36,37,38,39,40] and ECG analysis during cardiopulmonary resuscitation [41,42,43].

Arrhythmia classification performance of AI software can be measured using regular performance metrics, such as false positives, false negatives, true positives, true negatives, accuracy (which is the proportion of examples that were correctly classified), global accuracy, precision (which is the proportion of correct predictions among all the predictions of a certain class), recall (which is the proportion of examples of a certain class that have been predicted by the model as belonging to that class), confidence threshold, area under the receiver operating characteristic curve (AUROC), the area under the precision recall curve (AUPRC), accuracy (defined here as the fraction of correctly classified recordings), F-measure, and in some research challenges a concrete “challenge evaluation metric”. Usually, the metric of choice for most people in AI to measure the performance of neural networks is F1 score. F1 score for a certain class is the harmonic mean of its precision and recall; thus, it is an overall measure of the quality of a classifier. F1 score measure or the average of the F1 values from each classification type is the score used in the PhysioNet/Computing in Cardiology Challenge (CinC), which is an annual platform to gather international competitors for forced development of open-source AI applications for specific ECG diagnosis [44].

Those challenges try to solve relevant clinical problems, such as arrhythmia classification using different lead sets ranging from two to twelve ECG leads amongst the vast amount of twelve-lead ECG recordings in the PhysioNet CinC Challenges 2020 and 2021. They are presently known as the largest freely available repository of standard 12-lead ECG records and consistent annotations for 30 clinical diagnoses of cardiac abnormalities [45,46,47,48,49].

Those metrics are a simple way to track or compare the performances of different models dealing with the same dataset in controlled environments (binary or multi-class scenarios). Performance results in real-life scenarios may vary depending on the heterogeneity of new data and the ability to generalize as concrete algorithm, the input duration or environments dealing with multi-label classification scenarios. For multi-label scenarios, confusion matrix may be very useful to evaluate performance.

### 2.2. Structural Heart Disease

**Figure 2:**
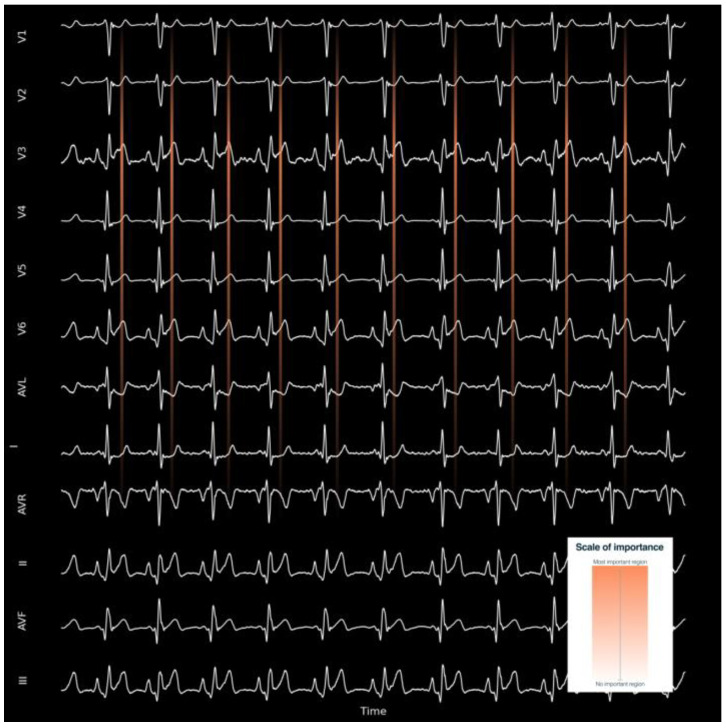
Example of a sensitivity map for understanding the region where the AI algorithm is focusing to predict an output. The most important region is in orange, and the least important regions are in black.

Left ventricular hypertrophy AI also has high testing accuracy, precision, sensitivity, and specificity of 0.96–0.97 [50]. AI algorithms have been shown to be capable of identifying left ventricular systolic dysfunction, not only with 12-lead ECG, but also with single-lead ECG obtained from smartwatches [51,52,53]. An AI-enabled smartwatch two-lead ECG can detect heart failure with reduced ejection fraction with reasonable performance: sensitivity, specificity, positive predictive value, and negative predictive value of 0.90, 0.86, 0.26, and 0.99, respectively [54]. These results suggest that left ventricular systolic dysfunction can be screened not only using a 12-lead ECG but also with a single-lead ECG performed by a wearable device employing the AI algorithm, thereby preventing irreversible disease progression and mortality [52]. The ability of ECG-based AI models to predict congestive heart failure and left ventricular systolic dysfunction has been confirmed in a meta-analysis [55]. The number of structural heart diseases that can be detected is increasing and already includes diseases, such as cardiac amyloidosis [56], pulmonary arterial hypertension [57], structural heart disease [58], significant aortic stenosis both with 12-lead and single-lead ECGs [59], and significant mitral regurgitation [60]. When performed, sensitivity maps can show where the algorithm is focusing, such as T wave of the precordial lead to determine the presence of significant aortic stenosis [59]. Figure 2 depicts how a sensitivity map (heatmaps) can show the neural network’s region of algorithm attention for determining the presence or absence of a cardiac pattern related with a concrete disease. Sensitivity maps may be useful for clinicians to understand the imbalance of the model weights and the regions of the ECG tracing on which the model focuses its attention to generate new ECG-based digital biomarkers. They help identify which features are most important for correct classification. For example, in object recognition in images, sensitivity maps can show which areas of the image are most relevant to determine whether the image shows a dog or a cat. The same techniques can be used to explain ECG models. There are many kinds of feature importance maps, generated by Grad-CAM [61], the SHapley Additive exPlanations (SHAP) method [62], global weights importance [63], among other methods. The latter demonstrated that the decision-making process in DenseNet noticeably corresponds to the cardiologists’ diagnostic point of view on the most prominent ECG characteristics for detection of atrial fibrillation, normal rhythms, other arrhythmia, and noise.

## 3. Risk Prediction and Integration with Clinical Variables

AI algorithms based solely on ECG data can be used for risk prediction. For instance, ECG data from implantable devices can accurately predict electrical remodeling, forecasting the progression from paroxysmal to persistent atrial fibrillation [64], and a deep learning model can identify patients at high risk for new-onset atrial fibrillation [65]. AI using clinical data without ECG is also effective in risk evaluation, such as in stroke prediction [66]. However, AI integration of ECG data with clinical variables is more effective to predict the risk of future cardiovascular events, including arrhythmias, myocardial infarction, stroke, and sudden cardiac death. Integrating ECG features with clinical variables (such as age, sex, comorbidities, previous cardiovascular events, and drugs) makes possible personalized risk estimates and is essential to increase accuracy. In fact, even when ECG evaluation is done by cardiologists, knowledge of the clinical characteristics of the individual patient on whom an ECG is ordered results in better ECG assessment and higher accuracy [67].

AI analyses of ECG of patients with ventricular fibrillation have been able to correlate spectral changes with acute cerebral injury, early prediction of mortality, and cerebral performance in comatose survivors after cardiac arrest [68], currently being validated in a multicenter study [69].

AI-based risk predictions have been shown to be accurate even in the general population. An ECG AI model has been shown to predict heart failure [70]. Using data from the Atherosclerosis Risk in Communities (ARIC) AI solely utilizing ECG achieved an area under the curve of 0.76, similar to the Framingham Heart Study Heart Failure risk calculator (0.78). The highest area under the curve (0.82) was obtained when using an ECG AI model output that integrated clinical variables (age, sex, race, body mass index, smoking status, coronary artery disease, diabetes mellitus, systolic blood pressure, and heart rate). AI provides accurate early detection of heart failure rehospitalization [71]. AI can rapidly identify severe hypo- and hyperkalemia; more importantly, it is an independent predictor for adverse outcomes [72]. AI can predict all-cause mortality with an area under the curve of 0.88 [73]; even within ECGs interpreted as ‘normal’ by a physician, the performance of the model in predicting one-year mortality is high (area under the curve = 0.85).

## 4. Monitoring of ECG Signals

Devices in real-time can alert clinicians or patients when significant changes occur according to timing, duration, and situation (exercise, sleep, etc.), laying a promising foundation for real-time computational clinical decision support systems regulated under the umbrella of software as a medical device. In addition, continuous ECG monitoring might detect changes in heart rate associated with medical interventions, such as hemodialysis [74].

**Figure 3:**
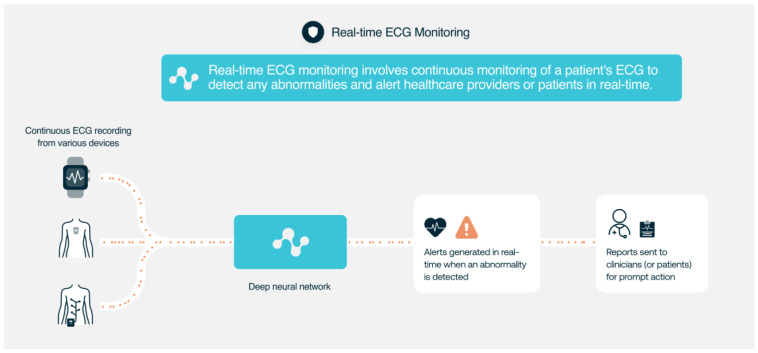
Use of AI ECG analysis for real-time ECG monitoring.

AI algorithms can use these ECG data to detect atrial fibrillation or bradycardia and notify patients to seek medical attention and can track ECG changes during exercise, sleep, or stress tests. AI can also be used to accurately monitor QT duration [75] or to classify premature ventricular contractions [76] (Figure 3). AI-based ECG analysis technology has many potential use cases involving different ECG devices and sensors. AI ECG has the potential to become what is currently known as a general-purpose AI system. As it has happened with some AI systems for language processing that now can be used as the foundation for several hundred applied models (e.g., chatbots, ad generation, decision assistants, spambots, translation, etc.), AI-based ECG technology can create in the coming years many solutions to support doctors dealing with cardiovascular and non-cardiovascular diseases in different environments with different intended uses of software as a medical device (SaMD).

Different commercial and research-based AI solutions are available for ECG monitoring or ECG analysis. Single-lead ECG wearables and smartwatches are increasingly used; some examples include Apple Watch, Kardia (AliveCor), Zio (iRhythm Technologies), BioHarness, Faros 360 (Bittium), CAM [77], Fitbit (Google), Willem (Idoven), and Scanwatch (Withings), among others. The AliveCor Kardia Mobile, a smartphone-based device equipped with two electrodes that enables remote participants to obtain a single-lead ECG, has also been analyzed with a validated AI algorithm demonstrating high specificity for arrhythmias detection. The device has been shown to have a sensitivity of 93% and a specificity of 84% for atrial fibrillation detection. The ZioPatch device uses an AI-powered algorithm to analyze continuous ECG recordings for up to 14 days and has been shown to improve the detection of arrhythmias and other cardiac events compared to conventional Holter monitoring [77,78]. The comparison for concrete use cases, such as to assess the accuracy of five direct-to-consumer wearable smart devices in identifying atrial fibrillation compared with a physician-interpreted 12-lead electrocardiogram with differences in the number of inconclusive tracings currently, diminishing the sensitivity and specificity of those wearables [79].

## 5. AI ECG Signal Processing for Improving Quality and Accuracy

AI ECG can enhance the quality and accuracy of ECG recordings by removing noise, artifacts, and interference. Portable and wearable devices often record ECG signals strongly corrupted with noise and artifacts; their signals are particularly attractive to implement AI algorithms. AI algorithms can detect QRS complex [80] and can discriminate high-quality and discard low-quality ECG excerpts of about 93%, only misclassifying around 5% of clean atrial fibrillation segments as noisy ones [81]. Recent studies have shown that these algorithms can be used to analyze smartwatch signals and to detect atrial fibrillation with only a small loss of sensitivity and specificity against a criterion-standard ECG. In fact, using the standard 12-lead ECG as the reference, the AI algorithm performance achieved a C statistic of 0.97 [82].

AI can be used to perform denoising block, acquiring the ECG signal from the patient and denoising it [83]. AI may significantly improve the quality of care in intensive care units by reducing the burden of false alarms [84]. AI can also extract features from ECG signals that are not visible to the human eye, such as heart rate variability.

A stacked denoising autoencoder based on deep neural networks (DNN) is a powerful technique used for classifying ECG heartbeats. The stacked denoising autoencoder (SDAE) is an artificial neural network consisting of multiple layers of encoding and decoding units. In the encoding stage, the input ECG signal is transformed into a compact representation, and in the decoding stage, the original signal is reconstructed from this compressed representation. Denoising is an essential pre-processing step to remove noise from the ECG signal before feeding it into the SDAE. This is because ECG signals are often corrupted by various types of noise, such as baseline wander, powerline interference, and muscle artifacts, which can significantly affect classification accuracy. The SDAE is trained using a large dataset of ECG signals with known labels. During training, the SDAE learns to extract relevant features from the input signal and accurately reconstruct the original signal. This process is repeated multiple times, and the network weights are adjusted each time to minimize the reconstruction error. Once trained, the SDAE can be used for ECG heartbeat classification. This is done by feeding a noisy ECG signal into the network and obtaining the compressed representation. This compact representation is then classified using a classifier to determine the type of heartbeat [85,86]. Liu, C. et al. published a dedicated special issue focused on detection of arrhythmia and noise from cardiovascular data in *Physiological Measurement* [87].

## 6. Diagnosis of Non-Cardiac Diseases

AI-based ECG analysis also has the potential to be used to detect or track non-cardiovascular disorders and to diagnose metabolic disorders, such as dyskalaemias [88], for non-invasive screening of hyperthyroidism [89], non-invasive anemia screening using raw ECG data [90], and cirrhosis [91], with similar levels of accuracy as the ones achieved detecting heart diseases, such as dilated cardiomyopathy [92]. AI ECG has the potential to be a tool to monitor disease status, cardiac hemodynamics, and drug therapeutic response as seen in preliminary data from patients with obstructive hypertrophic cardiomyopathy [93], where the AI ECG score likely reflect changes in the raw ECG waveform detectable by AI ECGs that correlate with hypertrophic cardiomyopathy disease pathophysiology and severity. As stated by Stéphane Hatem focusing on atrial fibrillation and stroke, we are probably at the edge of a new period where it will be possible to define an atrial vulnerability risk score for an individual. The information can be used to initiate a personalized medical strategy for upstream prevention. This is the goal of the H2020 consortium MAESTRIA created in 2020 [94].

AI-based analyses of ECG can rapidly exclude severe acute respiratory syndrome coronavirus 2 (SARS-CoV-2) infection with an under the curve for detection of acute COVID-19 infection of 0.78 with a negative predictive value of 99.2% [95].

## 7. Therapy Guidance and Treatment Optimization

AI can help in treatment guidance, assisting in patient selection, optimizing therapies, improving symptom-to-treatment times, and cost effectiveness. Some examples include earlier activation of code infarction in patients with ST-segment elevation and predicting the response to antiarrhythmic drugs or cardiac implantable device therapies, reduce the risk of cardiac toxicity associated with cancer immunotherapy and biological drugs, or predict the risk of drug-induced arrhythmias or long QT syndrome [96].

The accurate diagnosis of acute coronary occlusion is probably the main challenge for deep learning AI systems [97]. Currently, patients whose ECGs meet ST-segment elevation criteria are supposed to require immediate reperfusion. However, in 10–20% of them, ST-segment elevation is not due to acute coronary occlusion. The ST-segment elevation not associated with acute cardiac necrosis (LESTONNAC) [98] prospective registry is currently trying to validate an AI-based approach to identify these patients. In addition, about a quarter of “non-ST segment elevation” patients do have totally occluded coronary arteries and being able to identify them might prompt immediate reperfusion [75]. Implementation of an all-day, real-time AI-assisted remote detection of ST-segment elevation myocardial infarction on prehospital 12-lead ECGs has been shown to be feasible with a high diagnostic accuracy rate [99], making it possible to minimize delays in contact-to-treatment times for patients with ST-segment elevation. Even a limb six-lead ECG device can be used for detecting myocardial infarction with an area under the receiver operating characteristic curve of 0.88 [100]. An AI model has shown excellent performance in discriminating between control, ST-segment elevation myocardial infarction, and non-ST segment elevation myocardial infarction in a real-world sample of all-comers to an emergency department [101]. AI can be used to reduce door to ECG time in patients with ST-segment elevation myocardial infarction [102].

AI may be used to better classify patients into high- and low-risk subgroups and identify those most likely to derive benefit with the least side effects of medical therapy. AI might optimize patient selection for device therapy. Up to 40% of patients are non-responders to cardiac resynchronization therapy and AI models can improve patient selection by highlighting patients who are most likely to derive benefit from it [2]. AI models that use ECG data, such as heart rate variability to predict ventricular tachycardia, have good discrimination. These models can be used not only for the selection of patients that might benefit from implantable cardioverter defibrillation but also to warn patients that already have these devices, giving them the opportunity to pull over to park if driving or to find a place to sit or recline if walking [2].

## 8. Integration of ECG Data with Other Modalities

AI facilitates the integration of ECG data with other diagnostic modalities, mainly imaging techniques, genomics, proteomics, and biomarkers. The ability of AI and cloud high-performance computing to extract and analyze large volumes of data facilitates the integration not only with clinical variables but also with diagnostic techniques, different types of wearables, and even facial recognition [103]. For instance, patients with heart failure already benefit from a wide and expanding variety of sensor-enabled wearable, on- and near-body sensor technologies and implantable devices that generate massive amounts of data. The connectivity of all these devices has created opportunities for pooling data from multiple sensors—so-called interconnectivity. AI can provide new diagnostic, triage, risk-stratification, and disease management insights for the delivery of better and more personalized healthcare [104].

ECG and photoplethysmography data can provide valuable information on the cardiovascular system, including heart rate, blood flow, and arterial stiffness. By using this data in a deep learning regression model, the model can learn to estimate blood pressure values based on these physiological parameters accurately. Using ECG and photoplethysmography data for blood pressure estimation offers potential advantages due to their non-invasive and easy-to-obtain nature. ECG data can be recorded using electrodes placed on the skin, while photoplethysmography data can be obtained using wearable devices, such as smartwatches or fitness trackers. This enables continuous real-time monitoring of blood pressure without the need for invasive procedures.

Using deep learning regression models can also improve the accuracy of blood pressure estimation compared to traditional methods; deep learning regression models using ECG and photoplethysmography may be useful for the real-time estimation of systolic blood pressure and diastolic blood pressure values [105].

Using the impedance cardiogram (ICG) signal also known as cardioimpedance and the temporal location of the characteristic points B, C, and X provide crucial diagnostic information that can be used to determine cardiac output or stroke volume. However, there are several challenges to accurately identifying these characteristic points. The ICG signal is often noisy, and the morphology of the signal can vary significantly between patients. These factors make it challenging to identify the characteristic points accurately. Some methods have been demonstrated to be effective in identifying the characteristic points of the ICG signal. The process uses signal processing techniques and machine learning algorithms to determine the characteristic points with high accuracy and reliability. The proposed methods involve preprocessing the ICG signal using a bandpass filter to remove noise and extract relevant frequency components. Then, the signal is segmented into individuals’ heartbeats using a peak detection algorithm. Machine learning algorithms are finally used to classify each heartbeat and identify the characteristic points B, C, and X. The effectiveness of the proposed method has been confirmed in clinical pilot studies, demonstrating high accuracy and reliability in identifying the characteristic points of the ICG signal [106]. The technique may improve the accuracy of diagnostics in patients with heart failure, leading to better management and treatment of this condition.

Multimodal wearable sensing, leveraging the seismocardiogram, a sternal vibration signal associated with cardiomechanical activity, together with ECG offers a means to monitoring continuous stroke volume and may enable remote monitoring of cardiac function [107].

## 9. Improvement of Cost-Effectiveness

Cost effectiveness is relevant. Subclinical arrhythmias and cardiovascular disease are frequent. The fact that they might be detected by the widespread use of AI algorithms to detect conditions, such as atrial fibrillation, in large populations continuously wearing smartwatches would result in a substantial increase in new diagnoses. While there may be increased costs associated with the care of those patients, the potential reduction in cardiovascular events, such as strokes, could ultimately provide cost savings. There may be long-term clinical benefits of some cardiovascular disease screenings, such as in the case of atrial fibrillation or heart failure. Currently it is not cost effective to conduct systematic atrial fibrillation screening; however, AI-automated atrial fibrillation diagnosis has been achieved using a variety of rhythm modalities, including 12-lead ECGs, ambulatory ECGs, and photoplethysmography, and seems to improve cost effectiveness [108].

AI-ECG algorithms may also be used to expedite the evaluation of complex clinical cases in which multiple confounding factors are present and multiple diagnoses are under consideration [109] Studies focused on cost-effectiveness analysis of the use of AI in ECG analyses are scarce, and most integrate ECG data with other variables. The main message is that AI is effective for reducing cardiovascular disease burden; however, the fact that the populations used are different and have been performed in distinct health systems make extrapolations difficult. AI-based ECG could be an excellent screening tool and seems to have the potential to be cost effective [13]. AI analysis of ECG data might be more cost effective in some settings, such as hemodialysis, where ECG changes have been associated with prognosis. Prolonged PR, QRS, and QT intervals predict nonatherosclerotic cardiovascular events after the initiation of hemodialysis, while ECG criteria of left ventricular hypertrophy at the initiation of hemodialysis predicted atherosclerotic cardiovascular events in the short term [110]. In addition, AI models that use single-lead data yield a performance that is only slightly worse than the ones that use the full 12-lead data [111]. However, more research is needed to specifically examine the cost effectiveness of using AI in ECG.

## 10. Conclusions

**Figure 4:**
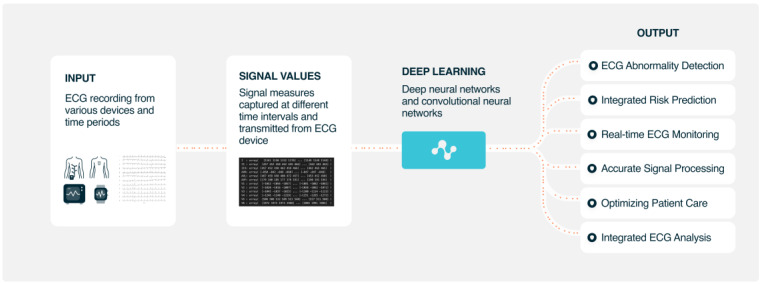
Possible use cases of AI ECG analysis in current clinical workflows.

AI-based ECG analyses can improve diagnosis and patient management. AI diagnostic performance is already similar to that of experienced cardiologists. AI ECG analyses seem to be cost effective and are able to reduce the rate of misdiagnosed computerized ECG interpretations and improve clinical efficiency, patient characterization, risk stratification, treatment selection, and optimization. As shown in Figure 4, AI may have many use cases to improve many current clinical processes where an ECG is involved to deal with cardiovascular diseases.

## 11. Future Directions

Most AI algorithms have been developed and tested on very controlled retrospective test and validation datasets; more work to test them prospectively and to assess their impact on real-world and real-time data is needed. Particularly, in the case of the possible applications of AI in selecting patients for medical and device therapies and optimizing care prospective testing in randomized controlled trials prior to clinical uptake seems mandatory. In the future, AI is expected to play an increasingly important role in ECG diagnosis and management, as more data become available and more sophisticated algorithms are developed. Physionet/Computing in Cardiology Challenge is an annual platform to gather international competitors from research centers and private companies for forced development of open-source AI, waveform, and rule-based algorithms for specific ECG diagnosis. The published databases for the challenge further become an open platform for many other research teams on AI [112].

## Figures and Tables

**Table 1:** Some areas where artificial intelligence algorithms can help clinicians in the field of electrocardiography.

Artificial Intelligence Use in Electrocardiography
Interpretation and detection of ECG abnormalities
Risk prediction integrated with or without clinical variables
Monitoring of ECG signals
ECG signal processing for improving quality and accuracy
Diagnosis of non-cardiac diseases
Therapy guidance and treatment optimization
Integration of ECG data with other modalities
Improvement of cost effectiveness

## Data Availability

Not applicable.

## References

[1] Somani S., Russak A.J., Richter F., Zhao S., Vaid A., Chaudhry F., De Freitas J.K., Naik N., Miotto R., Nadkarni G.N. (2021). Deep learning and the electrocardiogram: Review of the current state-of-the-art. Europace.

[2] Averbuch T., Sullivan K., Sauer A., A Mamas M., A Voors A., Gale C.P., Metra M., Ravindra N., Van Spall H.G.C. (2022). Applications of artificial intelligence and machine learning in heart failure. Eur. Heart J. -Digit. Health.

[3] Srivastava A., Pratiher S., Alam S., Hari A., Banerjee N., Ghosh N., Patra A. (2022). A deep residual inception network with channel attention modules for multi-label cardiac abnormality detection from reduced-lead ECG. Physiol. Meas..

[4] Teplitzky B.A., McRoberts M., Ghanbari H. (2020). Deep learning for comprehensive ECG annotation. Heart Rhythm.

[5] Wilson A., Saeed H., Pringle C., Eleftheriou I., A Bromiley P., Brass A. (2021). Artificial intelligence projects in healthcare: 10 practical tips for success in a clinical environment. BMJ Health Care Inform..

[6] Akoum N., Zelnick L.R., de Boer I.H., Hirsch I.B., Trence D., Henry C., Robinson N., Bansal N. (2019). Rates of Cardiac Rhythm Abnormalities in Patients with CKD and Diabetes. Clin. J. Am. Soc. Nephrol..

[7] Tsai D.-J., Tsai S.-H., Chiang H.-H., Lee C.-C., Chen S.-J. (2022). Development and Validation of an Artificial Intelligence Electrocardiogram Recommendation System in the Emergency Department. J. Pers. Med..

[8] Exarchos T.P., Tsipouras M.G., Exarchos C.P., Papaloukas C., Fotiadis D.I., Michalis L.K. (2007). A methodology for the automated creation of fuzzy expert systems for ischaemic and arrhythmic beat classification based on a set of rules obtained by a decision tree. Artif. Intell. Med..

[9] Malik J., Devecioglu O.C., Kiranyaz S., Ince T., Gabbouj M. (2021). Real-Time Patient-Specific ECG Classification by 1D Self-Operational Neural Networks. IEEE Trans. Biomed. Eng..

[10] Li P., Wang Y., He J., Wang L., Tian Y., Zhou T.-S., Li T., Li J.-S. (2016). High-Performance Personalized Heartbeat Classification Model for Long-Term ECG Signal. IEEE Trans. Biomed. Eng..

[11] Quartieri F., Marina-Breysse M., Pollastrelli A., Paini I., Lizcano C., Lillo-Castellano J.M., Grammatico A. (2022). Artificial intelligence augments detection accuracy of cardiac insertable cardiac monitors: Results from a pilot prospective observational study. Cardiovasc. Digit. Health J..

[12] Frohnert P.P., Gluliani E.R., Friedberg M., Johnson W.J., Tauxe W.N. (1970). Statistical Investigation of Correlations Between Serum Potassium Levels and Electrocardiographic Findings in Patients on Intermittent Hemodialysis Therapy. Circulation.

[13] Ahsan M., Siddique Z. (2022). Machine learning-based heart disease diagnosis: A systematic literature review. Artif. Intell. Med..

[14] Li G., Tan Z., Xu W., Xu F., Wang L., Chen J., Wu K. (2021). A particle swarm optimization improved BP neural network intelligent model for electrocardiogram classification. BMC Med. Inform. Decis. Mak..

[15] Zhang J., Liu A., Gao M., Chen X., Zhang X., Chen X. (2020). ECG-based multi-class arrhythmia detection using spatio-temporal attention-based convolutional recurrent neural network. Artif. Intell. Med..

[16] Haseena H.H., Mathew A.T., Paul J.K. (2009). Fuzzy Clustered Probabilistic and Multi Layered Feed Forward Neural Networks for Electrocardiogram Arrhythmia Classification. J. Med. Syst..

[17] Sayantan G., Kien P.T., Kadambari K.V. (2018). Classification of ECG beats using deep belief network and active learning. Med. Biol. Eng. Comput..

[18] Oh S.L., Ng E.Y., San Tan R., Acharya U.R. (2018). Automated diagnosis of arrhythmia using combination of CNN and LSTM techniques with variable length heart beats. Comput. Biol. Med..

[19] Taggar J.S., Coleman T., Lewis S., Heneghan C., Jones M. (2015). Accuracy of methods for diagnosing atrial fibrillation using 12-lead ECG: A systematic review and meta-analysis. Int. J. Cardiol..

[20] I Attia Z., A Noseworthy P., Lopez-Jimenez F., Asirvatham S.J., Deshmukh A.J., Gersh B.J., E Carter R., Yao X., A Rabinstein A., Erickson B.J. (2019). An artificial intelligence-enabled ECG algorithm for the identification of patients with atrial fibrillation during sinus rhythm: A retrospective analysis of outcome prediction. Lancet.

[21] Jo Y.-Y., Kwon J.-M., Jeon K.-H., Cho Y.-H., Shin J.-H., Lee Y.-J., Jung M.-S., Ban J.-H., Kim K.-H., Lee S.Y. (2021). Detection and classification of arrhythmia using an explainable deep learning model. J. Electrocardiol..

[22] Hannun A.Y., Rajpurkar P., Haghpanahi M., Tison G.H., Bourn C., Turakhia M.P., Ng A.Y. (2019). Cardiologist-level arrhythmia detection and classification in ambulatory electrocardiograms using a deep neural network. Nat. Med..

[23] Chang K.-C., Hsieh P.-H., Wu M.-Y., Wang Y.-C., Chen J.-Y., Tsai F.-J., Shih E.S., Hwang M.-J., Huang T.-C. (2020). Usefulness of Machine Learning-Based Detection and Classification of Cardiac Arrhythmias With 12-Lead Electrocardiograms. Can. J. Cardiol..

[24] Hughes J.W., Olgin J.E., Avram R., Abreau S.A., Sittler T., Radia K., Hsia H., Walters T., Lee B., Gonzalez J.E. (2021). Performance of a Convolutional Neural Network and Explainability Technique for 12-Lead Electrocardiogram Interpretation. JAMA Cardiol..

[25] Ribeiro A.H., Ribeiro M.H., Paixão G.M.M., Oliveira D.M., Gomes P.R., Canazart J.A., Ferreira M.P.S., Andersson C.R., Macfarlane P.W., Meira W. (2020). Automatic diagnosis of the 12-lead ECG using a deep neural network. Nat. Commun..

[26] Xu S.S., Mak M.-W., Cheung C.-C. (2018). Towards End-to-End ECG Classification with Raw Signal Extraction and Deep Neural Networks. IEEE J. Biomed. Health Inform..

[27] Zhu Z., Lan X., Zhao T., Guo Y., Kojodjojo P., Xu Z., Liu Z., Liu S., Wang H., Sun X. (2021). Identification of 27 abnormalities from multi-lead ECG signals: An ensembled SE_ResNet framework with Sign Loss function. Physiol. Meas..

[28] Xu Z., Guo Y., Zhao T., Zhao Y., Liu Z., Sun X., Xie G., Li Y. (2022). Abnormality classification from electrocardiograms with various lead combinations. Physiol. Meas..

[29] Fiorina L., Maupain C., Gardella C., Manenti V., Salerno F., Socie P., Li J., Henry C., Plesse A., Narayanan K. (2022). Evaluation of an Ambulatory ECG Analysis Platform Using Deep Neural Networks in Routine Clinical Practice. J. Am. Heart Assoc..

[30] Acharya U.R., Oh S.L., Hagiwara Y., Tan J.H., Adam M., Gertych A., San Tan R. (2017). A deep convolutional neural network model to classify heartbeats. Comput. Biol. Med..

[31] Puszkarski B., Hryniów K., Sarwas G. (2022). Comparison of neural basis expansion analysis for interpretable time series (N-BEATS) and recurrent neural networks for heart dysfunction classification. Physiol. Meas..

[32] Badertscher P., Lischer M., Mannhart D., Knecht S., Isenegger C., Lavallaz J.D.F.D., Schaer B., Osswald S., Kühne M., Sticherling C. (2022). Clinical validation of a novel smartwatch for automated detection of atrial fibrillation. Heart Rhythm. O2.

[33] Shah A.P., Rubin S.A. (2007). Errors in the computerized electrocardiogram interpretation of cardiac rhythm. J. Electrocardiol..

[34] Sabut S., Pandey O., Mishra B.S.P., Mohanty M. (2021). Detection of ventricular arrhythmia using hybrid time–frequency-based features and deep neural network. Phys. Eng. Sci. Med..

[35] Chang T.-Y., Chen K.-W., Liu C.-M., Chang S.-L., Lin Y.-J., Lo L.-W., Hu Y.-F., Chung F.-P., Lin C.-Y., Kuo L. (2022). A High-Precision Deep Learning Algorithm to Localize Idiopathic Ventricular Arrhythmias. J. Pers. Med..

[36] Shen C.P., Freed B.C., Walter D.P., Perry J.C., Barakat A.F., Elashery A.R.A., Shah K.S., Kutty S., McGillion M., Ng F.S. (2023). Convolution Neural Network Algorithm for Shockable Arrhythmia Classification Within a Digitally Connected Automated External Defibrillator. J. Am. Heart Assoc..

[37] Hajeb-M S., Cascella A., Valentine M., Chon K.H. (2021). Deep Neural Network Approach for Continuous ECG-Based Automated External Defibrillator Shock Advisory System During Cardiopulmonary Resuscitation. J. Am. Heart Assoc..

[38] Krasteva V., Ménétré S., Didon J.-P., Jekova I. (2020). Fully Convolutional Deep Neural Networks with Optimized Hyperparameters for Detection of Shockable and Non-Shockable Rhythms. Sensors.

[39] Irusta U., Aramendi E., Chicote B., Alonso D., Corcuera C., Veintemillas J., Larrea A., Olabarria M. (2019). Deep learning approach for a shock advise algorithm using short electrocardiogram analysis intervals. Resuscitation.

[40] Picon A., Irusta U., Álvarez-Gila A., Aramendi E., Alonso-Atienza F., Figuera C., Ayala U., Garrote E., Wik L., Kramer-Johansen J. (2019). Mixed convolutional and long short-term memory network for the detection of lethal ventricular arrhythmia. PLoS ONE.

[41] Jekova I., Krasteva V. (2021). Optimization of end-to-end convolutional neural networks for analysis of out-of-hospital cardiac arrest rhythms during cardiopulmonary resuscitation. Sensors.

[42] Gong Y., Wei L., Yan S., Zuo F., Zhang H., Li Y. (2023). Transfer learning based deep network for signal restoration and rhythm analysis during cardiopulmonary resuscitation using only the ECG waveform. Inf. Sci..

[43] Isasi I., Irusta U., Aramendi E., Eftestøl T., Kramer-Johansen J., Wik L. (2020). Rhythm Analysis during Cardiopulmonary Resuscitation Using Convolutional Neural Networks. Entropy.

[44] Clifford G.D., Liu C., Moody B., Lehman L.H., Silva I., Li Q., Johnson A.E., Mark R.G. AF classification from a short single lead ECG recording: The PhysioNet/Computing in Cardiology Challenge 2017. Proceedings of the 2017 Computing in Cardiology (CinC).

[45] Perez Alday E.A., Gu A., Shah A., Liu C., Sharma A., Seyedi S., Bahrami Rad A., Reyna M., Clifford G. (2022). Classi-Fication of 12-Lead ECGs: The PhysioNet/Computing in Cardiology Challenge 2020 (Version 1.0.2).

[46] Alday E.A.P., Gu A., Shah A.J., Robichaux C., Wong A.-K.I., Liu C., Liu F., Rad A.B., Elola A., Seyedi S. (2020). Classification of 12-lead ECGs: The PhysioNet/Computing in Cardiology Challenge 2020. Physiol. Meas..

[47] Ren Y., Liu F., Xia S., Shi S., Chen L., Wang Z. (2023). Dynamic ECG signal quality evaluation based on persistent homology and GoogLeNet method. Front Neurosci..

[48] Reyna M., Sadr N., Alday E.A.P., Gu A., Shah A.J., Robichaux C., Rad A.B., Elola A., Seyedi S., Ansari S. (2021). Will Two Do? Varying Dimensions in Electrocardiography: The PhysioNet/Computing in Cardiology Challenge 2021. Comput. Cardiol..

[49] Reyna M., Sadr N., Alday E.A.P., Gu A., Shah A.J., Robichaux C., Rad A.B., Elola A., Seyedi S., Ansari S. (2022). Issues in the automated classification of multilead ecgs using heterogeneous labels and populations. Physiol. Meas..

[50] Liu C.-W., Wu F.-H., Hu Y.-L., Pan R.-H., Lin C.-H., Chen Y.-F., Tseng G.-S., Chan Y.-K., Wang C.-L. (2023). Left ventricular hypertrophy detection using electrocardiographic signal. Sci. Rep..

[51] Attia Z.I., Kapa S., Lopez-Jimenez F., McKie P.M., Ladewig D.J., Satam G., Pellikka P.A., Enriquez-Sarano M., Noseworthy P.A., Munger T.M. (2019). Screening for cardiac contractile dysfunction using an artificial intelligence–enabled electrocardiogram. Nat. Med..

[52] Cho J., Lee B., Kwon J.-M., Lee Y., Park H., Oh B.-H., Jeon K.-H., Park J., Kim K.-H. (2020). Artificial Intelligence Algorithm for Screening Heart Failure with Reduced Ejection Fraction Using Electrocardiography. ASAIO J..

[53] Attia Z.I., Harmon D.M., Dugan J., Manka L., Lopez-Jimenez F., Lerman A., Siontis K.C., Noseworthy P.A., Yao X., Klavetter E.W. (2022). Prospective evaluation of smartwatch-enabled detection of left ventricular dysfunction. Nat. Med..

[54] Kwon J.-M., Jo Y.-Y., Lee S.Y., Kang S., Lim S.-Y., Lee M.S., Kim K.-H. (2022). Artificial Intelligence-Enhanced Smartwatch ECG for Heart Failure-Reduced Ejection Fraction Detection by Generating 12-Lead ECG. Diagnostics.

[55] Grün D., Rudolph F., Gumpfer N., Hannig J., Elsner L.K., von Jeinsen B., Hamm C.W., Rieth A., Guckert M., Keller T. (2021). Identifying Heart Failure in ECG Data with Artificial Intelligence—A Meta-Analysis. Front. Digit. Health.

[56] Grogan M., Lopez-Jimenez F., Cohen-Shelly M., Dispenzieri A., Attia Z.I., Ezzedine O.F.A., Lin G., Kapa S., Borgeson D.D., Friedman P.A. (2021). Artificial Intelligence–Enhanced Electrocardiogram for the Early Detection of Cardiac Amyloidosis. Mayo Clin. Proc..

[57] Tison G., Zhang J., Delling F.N., Deo R.C. (2019). Automated and Interpretable Patient ECG Profiles for Disease Detection, Tracking, and Discovery. Circ. Cardiovasc. Qual. Outcomes.

[58] Ulloa-Cerna A.E., Jing L., Pfeifer J.M., Raghunath S., Ruhl J.A., Rocha D.B., Leader J.B., Zimmerman N., Lee G., Steinhubl S.R. (2022). rECHOmmend: An ECG-Based Machine Learning Approach for Identifying Patients at Increased Risk of Undiagnosed Structural Heart Disease Detectable by Echocardiography. Circulation.

[59] Kwon J., Lee S.Y., Jeon K., Lee Y., Kim K., Park J., Oh B., Lee M. (2020). Deep Learning–Based Algorithm for Detecting Aortic Stenosis Using Electrocardiography. J. Am. Heart Assoc..

[60] Kwon J.-M., Kim K.-H., Akkus Z., Jeon K.-H., Park J., Oh B.-H. (2020). Artificial intelligence for detecting mitral regurgitation using electrocardiography. J. Electrocardiol..

[61] Makimoto H., Höckmann M., Lin T., Glöckner D., Gerguri S., Clasen L., Schmidt J., Assadi-Schmidt A., Bejinariu A., Müller P. (2020). Performance of a convolutional neural network derived from an ECG database in recognizing myocardial infarction. Sci. Rep..

[62] Zhang D., Yang S., Yuan X., Zhang P. (2021). Interpretable deep learning for automatic diagnosis of 12-lead electrocardiogram. iScience.

[63] Krasteva V., Christov I., Naydenov S., Stoyanov T., Jekova I. (2021). Application of Dense Neural Networks for Detection of Atrial Fibrillation and Ranking of Augmented ECG Feature Set. Sensors.

[64] Lillo-Castellano J.M., González-Ferrer J.J., Marina-Breysse M., Martínez-Ferrer J.B., Pérez-Álvarez L., Alzueta J., Martínez J.G., Rodríguez A., Rodríguez-Pérez J.C., Anguera I. (2019). Personalized monitoring of electrical remodelling during atrial fibrillation progression via remote transmissions from implantable devices. Europace.

[65] Raghunath S., Pfeifer J.M., Ulloa-Cerna A.E., Nemani A., Carbonati T., Jing L., Vanmaanen D.P., Hartzel D.N., Ruhl J.A., Lagerman B.F. (2021). Deep Neural Networks Can Predict New-Onset Atrial Fibrillation From the 12-Lead ECG and Help Identify Those at Risk of Atrial Fibrillation–Related Stroke. Circulation.

[66] Lip G.Y.H., Tran G., Genaidy A., Marroquin P., Estes C., Landsheftl J. (2021). Improving dynamic stroke risk prediction in non-anticoagulated patients with and without atrial fibrillation: Comparing common clinical risk scores and machine learning algorithms. Eur. Heart J. -Qual. Care Clin. Outcomes.

[67] Anh D., Krishnan S., Bogun F. (2006). Accuracy of electrocardiogram interpretation by cardiologists in the setting of incorrect computer analysis. J. Electrocardiol..

[68] Filgueiras-Rama D., Calvo C.J., Salvador-Montañés Ó., Cádenas R., Ruiz-Cantador J., Armada E., Rey J.R., Merino J., Peinado R., Pérez-Castellano N. (2015). Spectral analysis-based risk score enables early prediction of mortality and cerebral performance in patients undergoing therapeutic hypothermia for ventricular fibrillation and comatose status. Int. J. Cardiol..

[69] Palacios-Rubio J., Marina-Breysse M., Quintanilla J.G., Gil-Perdomo J.M., Juárez-Fernández M., Garcia-Gonzalez I., Rial-Bastón V., Corcobado M.C., Espinosa M.C., Ruiz F. (2018). Early prognostic value of an Algorithm based on spectral Variables of Ventricular fibrillAtion from the EKG of patients with suddEn cardiac death: A multicentre observational study (AWAKE). Arch. Cardiol. Mex..

[70] Akbilgic O., Butler L., Karabayir I., Chang P.P., Kitzman D.W., Alonso A., Chen L.Y., Soliman E.Z. (2021). ECG-AI: Electrocardiographic artificial intelligence model for prediction of heart failure. Eur. Heart J. -Digit. Health.

[71] Stehlik J., Schmalfuss C., Bozkurt B., Nativi-Nicolau J., Wohlfahrt P., Wegerich S., Rose K., Ray R., Schofield R., Deswal A. (2020). Continuous Wearable Monitoring Analytics Predict Heart Failure Hospitalization. Circ. Heart Fail..

[72] Lin C., Chau T., Shang H.-S., Fang W.-H., Lee D.-J., Lee C.-C., Tsai S.-H., Wang C.-H., Lin S.-H. (2022). Point-of-care artificial intelligence-enabled ECG for dyskalemia: A retrospective cohort analysis for accuracy and outcome prediction. NPJ Digit. Med..

[73] Raghunath S., Cerna A.E.U., Jing L., Vanmaanen D.P., Stough J., Hartzel D.N., Leader J.B., Kirchner H.L., Stumpe M.C., Hafez A. (2020). Prediction of mortality from 12-lead electrocardiogram voltage data using a deep neural network. Nat. Med..

[74] Rogovoy N.M., Howell S.J., Lee T.L., Hamilton C., Alday E.A.P., Kabir M.M., Zhang Y., Kim E.D., Fitzpatrick J., Monroy-Trujillo J.M. (2019). Hemodialysis Procedure–Associated Autonomic Imbalance and Cardiac Arrhythmias: Insights From Continuous 14-Day ECG Monitoring. J. Am. Heart Assoc..

[75] Maille B., Wilkin M., Million M., Rességuier N., Franceschi F., Koutbi-Franceschi L., Hourdain J., Martinez E., Zabern M., Gardella C. (2021). Smartwatch Electrocardiogram and Artificial Intelligence for Assessing Cardiac-Rhythm Safety of Drug Therapy in the COVID-19 Pandemic. The QT-logs study. Int. J. Cardiol..

[76] Mazidi M.H., Eshghi M., Raoufy M.R. (2022). Premature Ventricular Contraction (PVC) Detection System Based on Tunable Q-Factor Wavelet Transform. J. Biomed. Phys. Eng..

[77] Abdou A., Krishnan S. (2022). Horizons in Single-Lead ECG Analysis from Devices to Data. Front. Signal Process..

[78] Barrett P.M., Komatireddy R., Haaser S., Topol S., Sheard J., Encinas J., Fought A.J., Topol E.J. (2014). Comparison of 24-hour Holter Monitoring with 14-day Novel Adhesive Patch Electrocardiographic Monitoring. Am. J. Med..

[79] Mannhart D., Lischer M., Knecht S., Lavallaz J.D.F.D., Strebel I., Serban T., Vögeli D., Schaer B., Osswald S., Mueller C. (2023). Clinical Validation of 5 Direct-to-Consumer Wearable Smart Devices to Detect Atrial Fibrillation. JACC Clin. Electrophysiol..

[80] Kim J., Shin H. (2016). Simple and Robust Realtime QRS Detection Algorithm Based on Spatiotemporal Characteristic of the QRS Complex. PLoS ONE.

[81] Herraiz H., Martínez-Rodrigo A., Bertomeu-González V., Quesada A., Rieta J.J., Alcaraz R. (2020). A Deep Learning Approach for Featureless Robust Quality Assessment of Intermittent Atrial Fibrillation Recordings from Portable and Wearable Devices. Entropy.

[82] Tison G., Sanchez J.M., Ballinger B., Singh A., Olgin J.E., Pletcher M.J., Vittinghoff E., Lee E.S., Fan S.M., Gladstone R.A. (2018). Passive Detection of Atrial Fibrillation Using a Commercially Available Smartwatch. JAMA Cardiol..

[83] Deevi S.A., Kaniraja C.P., Mani V.D., Mishra D., Ummar S., Satheesh C. (2021). HeartNetEC: A deep representation learning approach for ECG beat classification. Biomed. Eng. Lett..

[84] Bollepalli S.C., Sevakula R.K., Au-Yeung W.M., Kassab M.B., Merchant F.M., Bazoukis G., Boyer R., Isselbacher E.M., Armoundas A.A. (2021). Real-Time Arrhythmia Detection Using Hybrid Convolutional Neural Networks. J. Am. Heart Assoc..

[85] Nurmaini S., Darmawahyuni A., Mukti A.N.S., Rachmatullah M.N., Firdaus F., Tutuko B. (2020). Deep Learning-Based Stacked Denoising and Autoencoder for ECG Heartbeat Classification. Electronics.

[86] Pravin C., Ojha V. A Novel ECG Signal Denoising Filter Selection Algorithm Based on Conventional Neural Networks. Proceedings of the 2020 19th IEEE International Conference on Machine Learning and Applications (ICMLA).

[87] Liu C., Lehman L., Moody B., Li Q., Clifford G. (2018). Focus on Detection of Arrhythmia and Noise from Cardiovascular Data. Physiol. Meas..

[88] Lou Y.-S., Lin C.-S., Fang W.-H., Lee C.-C., Wang C.-H. (2022). Development and validation of a dynamic deep learning algorithm using electrocardiogram to predict dyskalaemias in patients with multiple visits. Eur. Heart J. -Digit. Health.

[89] Choi B., Jang J.H., Son M., Lee M.S., Jo Y.Y., Jeon J.Y., Jin U., Soh M., Park R.W., Kwon J.M. (2022). Electrocardiographic biomarker based on machine learning for detecting overt hyperthyroidism. Eur. Heart J. -Digit. Health.

[90] Kwon J.-M., Cho Y., Jeon K.-H., Cho S., Kim K.-H., Baek S.D., Jeung S., Park J., Oh B.-H. (2020). A deep learning algorithm to detect anaemia with ECGs: A retrospective, multicentre study. Lancet Digit. Health.

[91] Ahn J.C., Attia Z.I., Rattan P., Mullan A.F., Buryska S., Allen A.M., Kamath P.S., Friedman P.A., Shah V.H., Noseworthy P.A. (2021). Development of the AI-Cirrhosis-ECG Score: An Electrocardiogram-Based Deep Learning Model in Cirrhosis. Am. J. Gastroenterol..

[92] Shrivastava S., Cohen-Shelly M., Attia Z.I., Rosenbaum A.N., Wang L., Giudicessi J.R., Redfield M., Bailey K., Lopez-Jimenez F., Lin G. (2021). Artificial Intelligence-Enabled Electrocardiography to Screen Patients with Dilated Cardiomyopathy. Am. J. Cardiol..

[93] Tison G.H., Siontis K.C., Abreau S., Attia Z., Agarwal P., Balasubramanyam A., Li Y., Sehnert A.J., Edelberg J.M., Friedman P.A. (2022). Assessment of Disease Status and Treatment Response with Artificial Intelligence−Enhanced Electrocardiography in Obstructive Hypertrophic Cardiomyopathy. J. Am. Coll. Cardiol..

[94] Hatem S.N., Cohen A. (2021). Atrial fibrillation and stroke: Are we looking in the right direction?. Cardiovasc. Res..

[95] Attia Z.I., Kapa S., Dugan J., Pereira N., Noseworthy P.A., Jimenez F.L., Cruz J., Carter R.E., DeSimone D.C., Signorino J. (2021). Rapid Exclusion of COVID Infection With the Artificial Intelligence Electrocardiogram. Mayo Clin. Proc..

[96] Prifti E., Fall A., Davogustto G., Pulini A., Denjoy I., Funck-Brentano C., Khan Y., Durand-Salmon A., Badilini F., Wells Q.S. (2021). Deep learning analysis of electrocardiogram for risk prediction of drug-induced arrhythmias and diagnosis of long QT syndrome. Eur. Heart J..

[97] McLaren J.T., Meyers H.P., Smith S.W. (2023). Kenichi Harumi Plenary Address at Annual Meeting of the International Society of Computers in Electrocardiology: “What Should ECG Deep Learning Focus on? The diagnosis of acute coronary occlusion!”. J. Electrocardiol..

[98] Martínez-Sellés M., Juárez M., Marina-Breysse M., Lillo-Castellano J.M., Ariza A. (2021). Rational and design of ST-segment elevation not associated with acute cardiac necrosis (LESTONNAC). A prospective registry for validation of a deep learning system assisted by artificial intelligence. J. Electrocardiol..

[99] Chen K.-W., Wang Y.-C., Liu M.-H., Tsai B.-Y., Wu M.-Y., Hsieh P.-H., Wei J.-T., Shih E.S.C., Shiao Y.-T., Hwang M.-J. (2022). Artificial intelligence-assisted remote detection of ST-elevation myocardial infarction using a mini-12-lead electrocardiogram device in prehospital ambulance care. Front. Cardiovasc. Med..

[100] Cho Y., Kwon J.-M., Kim K.-H., Medina-Inojosa J.R., Jeon K.-H., Cho S., Lee S.Y., Park J., Oh B.-H. (2020). Artificial intelligence algorithm for detecting myocardial infarction using six-lead electrocardiography. Sci. Rep..

[101] Gustafsson S., Gedon D., Lampa E., Ribeiro A.H., Holzmann M.J., Schön T.B., Sundström J. (2022). Development and validation of deep learning ECG-based prediction of myocardial infarction in emergency department patients. Sci. Rep..

[102] Su H.-Y., Tsai J.-L., Hsu Y.-C., Lee K.-H., Chang C.-S., Sun C.-K., Wang Y.-H., Chi S.-C., Hsu C.-W. (2021). A modified cardiac triage strategy reduces door to ECG time in patients with ST elevation myocardial infarction. Sci. Rep..

[103] Antoniades C., Asselbergs F.W., Vardas P. (2021). The year in cardiovascular medicine 2020: Digital health and innovation. Eur. Heart J..

[104] Bachtiger P., Plymen C.M., A Pabari P., Howard J.P., I Whinnett Z., Opoku F., Janering S., A Faisal A., Francis D.P., Peters N.S. (2020). Artificial Intelligence, Data Sensors and Interconnectivity: Future Opportunities for Heart Failure. Card. Fail. Rev..

[105] Li Y.-H., Harfiya L.N., Purwandari K., Lin Y.-D. (2020). Real-Time Cuffless Continuous Blood Pressure Estimation Using Deep Learning Model. Sensors.

[106] Karpiel I., Richter-Laskowska M., Feige D., Gacek A., Sobotnicki A. (2022). An Effective Method of Detecting Characteristic Points of Impedance Cardiogram Verified in the Clinical Pilot Study. Sensors.

[107] Ganti V.G., Gazi A.H., An S., Srivatsa A.V., Nevius B.N., Nichols C.J., Carek A.M., Fares M., Abdulkarim M., Hussain T. (2022). Wearable Seismocardiography-Based Assessment of Stroke Volume in Congenital Heart Disease. J. Am. Heart Assoc..

[108] Sivanandarajah P., Wu H., Bajaj N., Khan S., Ng F.S. (2022). Is machine learning the future for atrial fibrillation screening?. Cardiovasc. Digit. Health J..

[109] Harmon D.M., Witt D.R., Friedman P.A., Attia Z.I. (2021). Diagnosis and treatment of new heart failure with reduced ejection fraction by the artificial intelligence–enhanced electrocardiogram. Cardiovasc. Digit. Health J..

[110] Yamaguchi S., Hamano T., Oka T., Doi Y., Kajimoto S., Yasuda S., Shimada K., Matsumoto A., Sakaguchi Y., Inoue K. (2021). Electrocardiogram findings at the initiation of hemodialysis and types of subsequent cardiovascular events. Hypertens. Res..

[111] Chen T.-M., Huang C.-H., Shih E.S., Hu Y.-F., Hwang M.-J. (2020). Detection and Classification of Cardiac Arrhythmias by a Challenge-Best Deep Learning Neural Network Model. iScience.

[112] Goldberger A., Amaral L., Glass L., Hausdorff J., Ivanov P.C., Mark R., Mietus J.E., Moody G.B., Peng C.-K., Stanley H.E. (2000). PhysioBank, Phys-ioToolkit, and PhysioNet: Components of a new research resource for complex physiologic signals. Circulation.

